# Realization of Lasing Emission from One Step Fabricated WSe_2_ Quantum Dots

**DOI:** 10.3390/nano8070538

**Published:** 2018-07-17

**Authors:** Pengpeng Ren, Wenfei Zhang, Yiqun Ni, Di Xiao, Honghao Wan, Ya-Pei Peng, Ling Li, Peiguang Yan, Shuangchen Ruan

**Affiliations:** Shenzhen Key Laboratory of Laser Engineering, College of Optoelectronic Engineering, Shenzhen University, Shenzhen 518060, China; 2160190404@email.szu.edu.cn (P.R.); 18394667281@163.com (Y.N.); 2170285214@email.szu.edu.cn (D.X.); 2170285219@email.szu.edu.cn (H.W.); yppeng@szu.edu.cn (Y.-P.P.); liling@szu.edu.cn (L.L.); yanpg@szu.edu.cn (P.Y.); scruan@szu.edu.cn (S.R.)

**Keywords:** WSe_2_ quantum dots, Laser, Two-dimensional transition-metal dichalcogenides, photoluminenscence

## Abstract

Two-dimensional (2D) transition-metal dichalcogenides (TMDCs) quantum dots (QDs) are the vanguard due to their unique properties. In this work, WSe_2_ QDs were fabricated via one step ultrasonic probe sonication. Excitation wavelength dependent photoluminescence (PL) is observed from WSe_2_ QDs. Room-temperature lasing emission which benefits from 3.7 times enhancement of PL intensity by thermal treatment at ~470 nm was achieved with an excitation threshold value of ~3.5 kW/cm^2^ in a Fabry–Perot laser cavity. To the best of our knowledge, this is the first demonstration of lasing emission from TMDCs QDs. This indicates that TMDCs QDs are a superior candidate as a new type of laser gain medium.

## 1. Introduction

Nanoluminescent materials have always been the hotspot of scientific research [1,2,3]. Among these, pioneering work on layered materials has been done to explore the potential of layered materials in diverse applications [4]. Two-dimensional (2D) transition-metal dichalcogenides (TMDCs), especially, have attracted significant attention because of their unique properties [5,6,7,8,9,10]. TMDCs quantum dots (QDs) have also been studied intensively as future optoelectronic materials owing to their dramatic properties [4,11,12,13] and widespread applications, such as fluorescent imaging, biomedical imaging, biological sensing [14], light emitting devices [15], photocatalytic, and hydrogen generation [16] et al. [17,18]. TMDCs systems have been demonstrated to exhibit distinctive photoluminescence (PL) characteristics [19,20,21,22] because of their direct energy band gap. Increasing PL by decreasing the number of layers of materials was reported by Wang et al. [23]. Sefaattin Tongay et al., successfully exfoliated single-layer MoSe_2_ for the first time which showed good thermal stability with a 1.55 eV direct bandgap [24]. After that, PL from 2D TMDCs have been widely studied due to their unique photoluminescence properties. Hao et al., realized both near infrared-to- near infrared up-conversion and down-conversion light-emissions by fabricating a novel 2D system of Er-doped MoS_2_ layered nanosheets [25]. Chen et al. significantly enhanced the Raman signal and PL of various 2D materials by using a two-layer nanocavity which comprised a non-absorbing spacer deposited on a flat metal reflector [26]. TMDCs QDs also exhibit excellent PL properties which profit from quantum confinement effect and some of the inherent merits of the 2D materials. X. Bai et al. fabricated ultrasmall WS_2_ QDs with catalytic properties and PL characteristics at visible wavelength [27]. Ultraviolet irradiation-induced oxidative etching in an aqueous solution to prepare photoluminescent MoS_2_ and WS_2_ QDs was reported by X. Lu et al. [28]. However, all the reported studies were just focused on normal PL from TMDCs QDs. Stimulated emission (i.e., lasing emission) from TMDCs QDs has not yet been reported. The realization of lasing emission is proof of excellent optical properties of QD materials. Perovskite QDs [29,30] and carbon nanodots [31,32] have already achieved lasing emission due to its distinct optical properties.

## 2. Experiment

WSe_2_ QDs were fabricated by using a method of probe sonication. Commercial WSe_2_ powder with an average particle size of 10–20 μm was dispersed in 50 mL Polyethylene Glycol 400 (PEG_400_). Then, the mixture was sonicated for 12 h in the sonicator. In this process, the WSe_2_ powder was crushed into QDs. After that, the suspension was centrifuged at the speed of 10,000 rpm for 10 min. After that, WSe_2_ QDs in PEG_400_ were collected from the supernatant. Finally, the as-prepared WSe_2_ QDs in PEG_400_ was placed inside a temperature regulated oven for thermal treatment at different temperatures.

The morphology of the WSe_2_ QDs was characterized by a JEOL JEM-2100F high-resolution transmission electron microscope (HR-TEM, Tokyo, Japan). Raman measurements were conducted on a Horiba-JY T64000 system (Horiba, Tokyo, Japan) using backscattering geometry; the incident laser power was 65 mW, and the laser wavelength was 514.5 nm with spot size about 2 μm. PL spectra were measured by a HORIBA iHR320 spectrometer (Minami-ku, Kyoto, Japan).The lasing characteristics of WSe_2_ QDs were studied by third harmonic generation from a neodymium-doped yttrium aluminum garnet (Nd:YAG) pulsed laser (355 nm, 10 Hz, Continuum Surelite, San Jose, CA, USA) with an optical parameter oscillator (Continuum Horizon, San Jose, CA, USA) to expand the Nd:YAG laser to different excitation wavelengths. The laser beam was focused onto the sample by an optical lens with a focal length of 50 mm and laser spot diameter of 125 μm. All of the measurements were conducted at room temperature.

## 3. Results and Discussions

**Figure 1:**
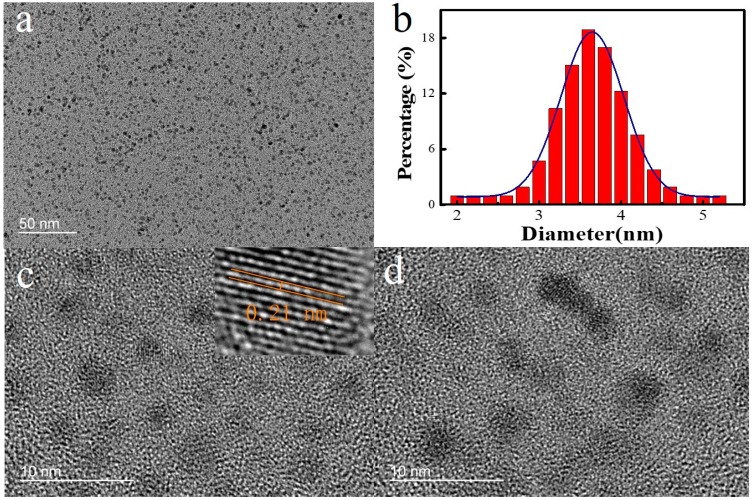
(**a**) Transmission electron microscopy (TEM) image, (**b**) Size distribution, (**c**,**d**) High-resolution transmission electron microscope (HR-TEM) image of the WSe_2_ QDs. Inset of (**c**): partial enlarged detail.

Ultrasonic probe sonication has been proved to be an efficient method to fabricate TMDCs QDs in liquid solvent because of the process of cavitation [11]. The morphology of the as-prepared WSe_2_ QDs shows a uniform distribution in the transmission electron microscopy (TEM) analysis. Figure 1a and b shows that the size of WSe_2_ QDs is distributed between 2 nm and 6 nm with an average size of 3.6 nm. The size distributions of WSe_2_ QDs exhibits a Gaussian profiler. (Figure 1b). Figure 1c,d are the HR-TEM of the WSe_2_ QDs. The lattice spacing, which is measured from an HR-TEM in the inset of Figure 1c of the WSe_2_ QDs which correspond to the (104) orientation, is measured to be about 0.21 nm [33]. These measurements indicate that the TMDCs QDs are highly crystalline in nature.

**Figure 2:**
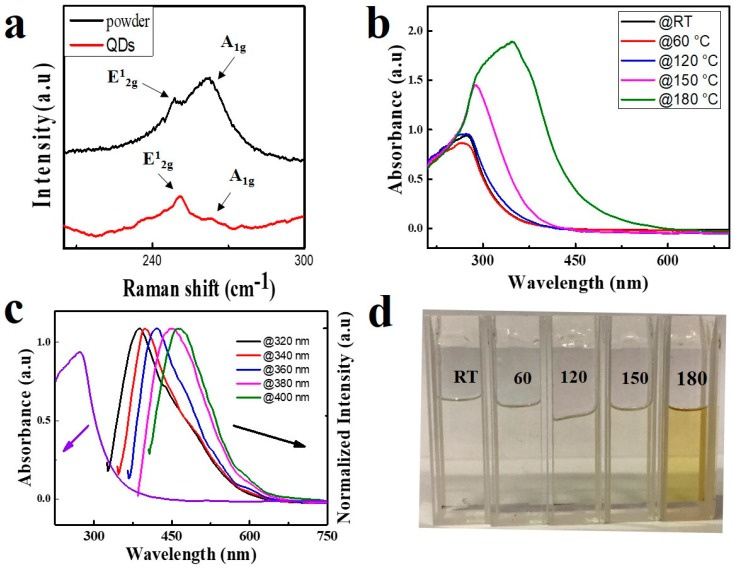
(**a**) Raman spectra of WSe_2_ quantum dots (QDs) (**b**) Ultraviolet-visible absorption spectrum of WSe_2_ QDs at different thermal treatment temperatures for 16 h. (**c**) Ultraviolet-visible light absorption spectra of WSe_2_ QDs and photoluminescence (PL) spectra of WSe_2_ QDs at different excitation wavelengths. (**d**) Photographs of the WSe_2_ QDs solution with different thermal treatment temperatures.

Figure 2a plots the Raman spectra of the WSe_2_ QDs and the WSe_2_ powder. A peak located at around 250 cm^−1^ can be seen clearly which represents E^1^_2g_ and A_1g_ peak of the WSe_2_ powder. Similarly, the same Raman signal can be found in that of WSe_2_ QDs [34]. This implies that the structure of the WSe_2_ QDs is similar to their powder. To further investigate the optical property of WSe_2_ QDs, ultraviolet-visible light absorption spectra were recorded for the as-prepared WSe_2_ QDs as shown in Figure 2b. Strong absorption from 250 to 400 nm is observed with an absorption peak located at 270 nm. Therefore, excitation wavelengths shorter than 400 nm are suitable for WSe_2_ QDs PL studies. Excitation wavelength dependent PL is also observed from WSe_2_ QDs. As shown in Figure 2c, the emission peak wavelength of WSe_2_ QDs redshift from 386 to 465 nm when excitation wavelength varies from 320 to 400 nm. The emission peak wavelengths of WSe_2_ QDs are located at the blue-violet area, which is significantly shorter than that of WSe_2_ bulk (i.e., located at the near-infrared area) [35]. This is due to the band broadening casing by quantum effects similar to some other QDs materials.

Intensive studies have been done on TMDCs QDs since the first discovery of their PL. However, most of the previous works were focused on normal PL only rather than stimulated emission from TMDCs QDs. This is because lower PL intensity and high optical loss hinder the application of TMDCs QDs in stimulated emission. In this work, the thermal treatment method is used to improve the PL intensity of the WSe_2_ QDs. Figure 2b plots the absorption spectrum of WSe_2_ QDs at different thermal treatment temperatures for 16 h. It is shown that the absorption at near ultraviolet increases with the increase of temperatures. Simultaneously, the absorption peak of the sample redshifts from 270 to 350 nm with temperature increases from room temperature to 180 °C. This phenomenon can also be confirmed by a change in color of the WSe_2_ QDs solutions. Figure 2d shows photographs of the WSe_2_ QDs solution with different thermal treatment temperatures. It can be seen that the color of WSe_2_ QDs solutions varies from colorless to pale yellow. This coincides with the ultraviolet-visible spectra.

**Figure 3:**
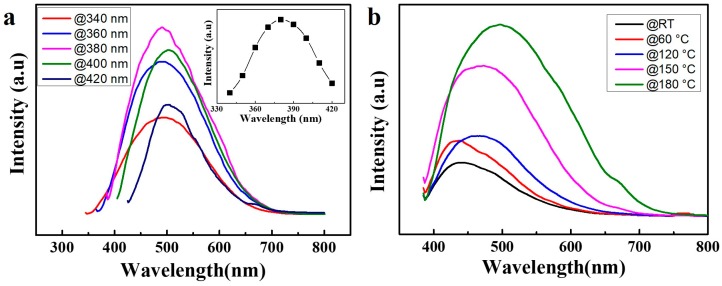
(**a**) PL spectra of WSe_2_ QDs after thermal treatment temperature of 180 °C. The inset is the excitation spectra of WSe_2_ QDs after thermal treatment temperature of 180 °C for 16 h. (**b**) PL spectrum of as-prepared samples at different thermal treatment temperatures at 380 nm excitation.

Figure 3a shows that the emission peak wavelength of WSe_2_ QDs (thermal treatment temperature of 180 °C) redshift from 477 to 505 nm when the excitation wavelength varies from 340 to 420 nm with a maximum emission at 380 nm excitation. The highest PL intensity is obtained at 380 nm excitation as is shown in the inset of Figure 3a. As a result, 380 nm is chosen as the excitation light source in the following studies. The Figure 3b shows the PL spectrum of as-prepared samples at different thermal treatment temperatures. The emission peak of WSe_2_ QDs redshifts from around 430 nm to 470 nm with increasing temperatures at 380 nm excitation. Most importantly, the intensity of PL can be enhanced by increasing the thermal treatment temperature. PL enhancement of as high as 3.7 times can be achieved by thermal treatment of WSe_2_ QDs at 180 °C.

**Figure 4:**
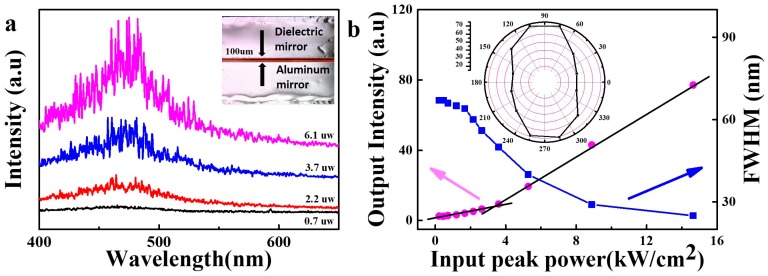
(**a**) Emission spectra versus different excitation power. The inset is the photograph of the designed microcavity (**b**) The output intensity and full width at half-maximum (FWHM) of the emission spectra versus input peak power. The inset polar patterns plot the lasing intensity at each given polarization state.

A Fabry-Perot laser cavity, which utilizes WSe_2_ QDs as laser gain medium, was fabricated by sandwiching a layer of WSe_2_ QDs in PEG_400_ (after thermal treatment at 180 °C for 16 h) between an aluminum mirror and a dielectric mirror. A 380 nm laser, which is the optimized excitation wavelength for WSe_2_ QDs, was chosen as the pump source. The inset of Figure 4a shows a photograph of the designed microcavity. The WSe_2_ QDs solution film, about 100 μm thick, was formed between an aluminum mirror and dielectric mirror. The mirrors are used to improve the longitudinal confinement of light and achieve optical feedback along the laser microcavity. The laser beam was focused onto a spot of 125 μm in diameter on the WSe_2_ QDs film through the dielectric mirror. Laser emission is detected from the side of the dielectric mirror perpendicular to the mirror plane. A plot of emission spectra of WSe_2_ QDs laser at room temperature versus different excitation power is shown in the Figure 4a. A broad spontaneous emission band centered at ~470 nm is observed when the excitation power is below an excitation threshold value of ~3.5 kW/cm^2^ as shown in Figure 4b. More sharp peaks emerge from the emission spectra with increasing pump power. Meanwhile, the full width at half-maximum (FWHM) of the spectrum also rapidly decreased from 67 nm to 25 nm. Such phenomenon indicates that the stimulated emission has played a leading role. Due to the coherent optical feedback provided by the WSe_2_ QDs to form the closed light loop path, the sharp peaks represent the realization of lasing. However, it can be observed from Figure 4a that the laser mode is distributed randomly. This is because a certain amount of heat is generated when the cavity is excited by the excitation light. Under the thermal perturbation, the liquid solution produces an uneven distribution of refractive index which leads to light scattering. The polarization character of the laser is also studied. The linear polarizer demonstrates the anisotropic properties of lasing emissions. The degree of polarization, defined as P = (I_1_ − I_2_)/(I_1_ + I_2_), is estimated, where I_1_ and I_2_ are the intensity of lasing emission with polarization at two different directions respectively. As shown in the inset of Figure 4b, the lasing emission of the WSe_2_ QDs is polarized, with the degree of polarization as P = 27.6%.

## 4. Conclusions

In this study, WSe_2_ QDs were fabricated via one step ultrasonic probe sonication in PEG_400_ [11,36]. Fluorescence enhancement as high as 3.7 times was achieved by thermal treatment of WSe_2_ QDs in PEG_400_. A Fabry-Perot laser cavity, which utilize WSe_2_ QDs as laser gain medium was fabricated by sandwiching a layer of WSe_2_ QDs in PEG_400_ between an aluminum mirror and a dielectric mirror. Lasing emission was achieved from the Fabry-Perot laser cavity by optical pumping at 380 nm. A laser threshold of ~3.5 kW/cm^2^ was recorded. FWHM of the lasing spectrum which narrowed from 67 nm to 25 nm was observed for the first time. This demonstration of lasing emission expands TMDCs QDs into a new application area.

## References

[1] Repp S., Erdem E. (2016). Controlling the exciton energy of zinc oxide (ZnO) quantum dots by changing the confinement conditions. Spectrochim. Acta A Mol. Biomol. Spectrosc..

[2] Repp S., Weber S., Erdem E. (2016). Defect evolution of nonstoichiometric ZnO quantum dots. J. Phys. Chem. C.

[3] Erdem E. (2017). Defect induced p-type conductivity in zinc oxide at high temperature: Electron paramagnetic resonance spectroscopy. Nanoscale.

[4] Valappil M.O., Anil A., Shaijumon M., Pillai V.K., Alwarappan S. (2017). A single-step electrochemical synthesis of luminescent WS_2_ quantum dots. Chemistry.

[5] Zhao Y.H., Yang F., Wang J., Guo H., Ji W. (2015). Continuously tunable electronic structure of transition metal dichalcogenides superlattices. Sci. Rep..

[6] Osada M., Sasaki T. (2009). Exfoliated oxide nanosheets: New solution to nanoelectronics. J. Mater. Chem..

[7] Wang Y., Ta V.D., Gao Y., He T.C., Chen R., Mutlugun E., Demir H.V., Sun H.D. (2014). Stimulated emission and lasing from CdSe/CdS/ZnS core-multi-shell quantum dots by simultaneous three-photon absorption. Adv. Mater..

[8] Yu T., Lim B., Xia Y. (2010). Aqueous-phase synthesis of single-crystal ceria nanosheets. Angew. Chem..

[9] Mak K.F., Shan J. (2016). Photonics and optoelectronics of 2D semiconductor transition metal dichalcogenides. Nat. Photon..

[10] Wang Q.H., Kalantar-Zadeh K., Kis A., Coleman J.N., Strano M.S. (2012). Electronics and optoelectronics of two-dimensional transition metal dichalcogenides. Nat. Nanotech..

[11] Bayat A., Saievar-Iranizad E. (2017). Synthesis of blue photoluminescent WS_2_ quantum dots via ultrasonic cavitation. J. Lumin..

[12] Kapatel S., Mania C., Sumesh C.K. (2017). Salt assisted sonochemical exfoliation and synthesis of highly stable few-to-monolayer WS_2_ quantum dots with tunable optical properties. J. Mater. Sci.-Mater. Electron..

[13] Long H., Tao L., Chiu C.P., Tang C.Y., Fung K.H., Chai Y., Tsang Y.H. (2016). The WS_2_ quantum dot: Preparation, characterization and its optical limiting effect in polymethylmethacrylate. Nanotechnology.

[14] Yan Y., Zhang C., Gu W., Ding C., Li X., Xian Y. (2016). Facile synthesis of water-soluble WS_2_ quantum dots for turn-on fluorescent measurement of lipoic acid. J. Phys. Chem. C.

[15] Ghorai A., Bayan S., Gogurla N., Midya A., Ray S.K. (2017). Highly luminescent WS_2_ quantum dots/ZnO heterojunctions for light emitting devices. ACS Appl. Mater. Interfaces.

[16] Xu S., Li D., Wu P. (2015). One-pot, facile, and versatile synthesis of monolayer MoS_2_/WS_2_ quantum dots as bioimaging probes and efficient electrocatalysts for hydrogen evolution reaction. Adv. Funct. Mater..

[17] Zhao X., Ma X., Sun J., Li D., Yang X. (2016). Enhanced catalytic activities of surfactant-assisted exfoliated WS(2) nnanodots for hydrogen evolution. ACS Nano.

[18] Zhou L., Yan S., Wu H., Song H., Shi Y. (2017). Facile sonication synthesis of WS2 quantum dots for photoelectrochemical performance. Catalysts.

[19] Tonndorf P., Schmidt R., Böttger P., Zhang X., Börner J., Liebig A., Albrecht M., Kloc C., Gordan O., Zahn D.R.T. (2013). Photoluminescence emission and raman response of monolayer MoS_2_, MoSe_2_, and WSe_2_. Opt. Express.

[20] Park J., Lee W., Choi T., Hwang S.H., Myoung J.M., Jung J.H., Kim S.H., Kim H. (2015). Layer-modulated synthesis of uniform tungsten disulfide nanosheet using gas-phase precursors. Nanoscale.

[21] Song Q., Liu L., Xiao S., Zhou X., Wang W., Xu L. (2006). Unidirectional high intensity narrow-linewidth lasing from a planar random microcavity laser. Phys. Rev. Lett..

[22] Ouyang Q., Zeng S., Jiang L., Hong L., Xu G., Dinh X.Q., Qian J., He S., Qu J., Coquet P. (2016). Sensitivity enhancement of transition metal dichalcogenides/silicon nanostructure-based surface plasmon resonance biosensor. Sci. Rep..

[23] Splendiani A., Sun L., Zhang Y., Li T., Kim J., Chim C.Y., Galli G., Wang F. (2010). Emerging photoluminescence in monolayer MoS_2_. Nano Lett..

[24] Tongay S., Zhou J., Ataca C., Lo K., Matthews T.S., Li J., Grossman J.C., Wu J. (2012). Thermally driven crossover from indirect toward direct bandgap in 2D semiconductors: MoSe_2_ versus MoS_2_. Nano Lett..

[25] Bai G., Yuan S., Zhao Y., Yang Z., Choi S.Y., Chai Y., Yu S.F., Lau S.P., Hao J. (2016). 2D layered materials of rare-earth er-doped MoS_2_ with NIR-to-NIR down- and up-conversion photoluminescence. Adv. Mater..

[26] Lee Y.C., Tseng Y.C., Chen H.L. (2016). Single type of nanocavity structure enhances light outcouplings from various two-dimensional materials by over 100-fold. ACS Photon..

[27] Bai X., Wang J., Mu X., Yang J., Liu H., Xu F., Jing Y., Liu L., Xue X., Dai H. (2017). Ultrasmall WS_2_ quantum dots with visible fluorescence for protection of cells and animal models from radiation-induced damages. ACS Biomater. Sci. Eng..

[28] Lu X., Wang R., Hao L., Yang F., Jiao W., Peng P., Yuan F., Liu W. (2016). Oxidative etching of MoS_2_/WS_2_ nanosheets to their QDs by facile UV irradiation. Phys. Chem. Chem. Phys..

[29] Tang X., Hu Z., Chen W., Xing X., Zang Z., Hu W., Qiu J., Du J., Leng Y., Jiang X. (2016). Room temperature single-photon emission and lasing for all-inorganic colloidal perovskite quantum dots. Nano Energy.

[30] Wang Y., Li X., Song J., Xiao L., Zeng H., Sun H. (2015). All-Inorganic colloidal perovskite quantum dots: A new class of lasing materials with favorable characteristics. Adv. Mater..

[31] Zhang W.F., Zhu H., Yu S.F., Yang H.Y. (2012). Observation of lasing emission from carbon nanodots in organic solvents. Adv. Mater..

[32] Qu S., Liu X., Guo X., Chu M., Zhang L., Shen D. (2014). Amplified spontaneous green emission and lasing emission from carbon nanoparticles. Adv. Funct. Mater..

[33] Zhang X., Lai Z., Liu Z., Tan C., Huang Y., Li B., Zhao M., Xie L., Huang W., Zhang H. (2015). A facile and universal top-down method for preparation of monodisperse transition-metal dichalcogenide nanodots. Angew. Chem..

[34] Li H., Wu J., Yin Z., Zhang H. (2014). Preparation and applications of mechanically exfoliated single-layer and multilayer MoS(2) and WSe(2) nanosheets. Acc. Chem. Res..

[35] Karfa P., Madhuri R., Sharma P.K. (2017). Multifunctional fluor escent chalcogenide hybrid nanodots (MoSe_2_:CdS and WSe_2_:CdS) as electro catalyst (for oxygen reduction/oxygen evolution reactions) and sensing probe for lead. J. Mater. Chem. A.

[36] Hernandez Y., Nicolosi V., Lotya M., Blighe F.M., Sun Z., De S., McGovern I.T., Holland B., Byrne M., Gun’ko Y.K. (2008). High-yield production of graphene by liquid-phase exfoliation of graphite. Nat. Nanotech..

